# Examining the impacts of public transit on healthy aging through a natural experiment: study protocols and lessons learned from the Active El Paso project

**DOI:** 10.3389/fpubh.2023.1132190

**Published:** 2023-07-25

**Authors:** Wei Li, Chanam Lee, Sinan Zhong, Minjie Xu, Samuel D. Towne Jr, Xuemei Zhu, Sungmin Lee, Suojin Wang, Rafael Aldrete, Eufemia B. Garcia, Leah Whigham, Ashley M. Toney, Jorge Ibarra, Marcia G. Ory

**Affiliations:** ^1^Department of Landscape Architecture and Urban Planning, School of Architecture, Texas A&M University, College Station, TX, United States; ^2^Center for Health Systems and Design, Texas A&M University, College Station, TX, United States; ^3^Center for Housing and Urban Development, Texas A&M University, College Station, TX, United States; ^4^Texas A&M Transportation Institute, Austin and El Paso, TX, United States; ^5^Department of Environmental and Occupational Health, School of Public Health, Texas A&M University, College Station, TX, United States; ^6^School of Global Health Management and Informatics, University of Central Florida, Orlando, FL, United States; ^7^Disability, Aging, and Technology Cluster, University of Central Florida, Orlando, FL, United States; ^8^Southwest Rural Health Research Center, Texas A&M University, College Station, TX, United States; ^9^Center for Community Health and Aging, Texas A&M University, College Station, TX, United States; ^10^Department of Architecture, School of Architecture, Texas A&M University, College Station, TX, United States; ^11^Department of Statistics, College of Arts and Sciences, Texas A&M University, College Station, TX, United States; ^12^Colonias Program, School of Architecture, Texas A&M University, College Station, TX, United States; ^13^Center for Community Health Impact and Department of Health Promotion and Behavioral Sciences, School of Public Health, The University of Texas Health Science Center at Houston, El Paso, TX, United States

**Keywords:** natural experiment, healthy aging, public transit, obesity, physical activity, public health, community health

## Abstract

This paper describes protocols and experiences from a seven-year natural-experiment study in El Paso, Texas, a border city of predominantly Latino/Hispanic population. The study focuses on how Bus Rapid Transit (BRT) impacts physical activity and thus plays a role in alleviating obesity and related chronic diseases that impact healthy aging. Our protocols describe a longitudinal and case-comparison study, which compared residents exposed to new BRT stations with those who were not. This paper also introduces lessons and experiences to overcome the following challenges: delays in the BRT opening (the main intervention), the COVID-19 pandemic, methodological challenges, participant recruitment and retention, and predatory survey takers. Our transdisciplinary approach was pivotal in addressing these challenges. We also proposed and tested multi-level intervention strategies to reduce modifiable barriers to transit use. Our most important takeaway for researchers, practitioners, and policy makers is the importance of being flexible and ready to adapt to new circumstances. Future natural-experiment researchers need to become more versatile in an increasingly volatile and uncertain world.

## 1. Introduction

This paper describes protocols and experiences from a seven-year natural-experiment study in El Paso, Texas, a US-Mexico border city with a predominantly Latino/Hispanic population. El Paso is the sixth-largest city in Texas with 81.50% of its population being of Hispanic origin (1). The median household income in El Paso, Texas is $48,866, with 50.90% of its households with less than $50,000 annual income (1).

Natural experiments have been increasingly used in observational studies across many disciplines to establish causality between complex real-world events and health-related outcomes (2), especially when traditional randomized clinical trials are not feasible. For example, planning researchers have conducted natural-experiment studies to investigate the impacts of changes in built environments (e.g., recreational destinations, transportation facilities) and planning policies on human behaviors (3, 4). Our study focuses on how Bus Rapid Transit (BRT) impacts physical activity (PA), motivated by the fact that the inadequate level of PA is a major public health risk factor associated with many chronic conditions and illnesses that impact population health across the life-course.

### 1.1. Obesity and physical activity

Obesity is a major risk factor for the onset or exacerbation of many chronic conditions such as cancer, diabetes, and/or heart disease (5). Globally, overweight and obesity are recognized as the 5^th^ leading cause of death (6). In the US, with an annual estimated cost of nearly $173 billion dollars for medical care alone, as many as 400,000 deaths each year have been attributed to obesity (7–11). US national data showed that Latino/Hispanic adults have a considerably higher rate of obesity compared to non-Latino/Hispanic White adults (12). Residents in low-income, minority communities suffer from a high prevalence of obesity (13, 14), cardiovascular disease (15, 16), and mental health disorders (17, 18), which have all been associated with physical inactivity. Low-income and minority populations are also more vulnerable to obesity and related illnesses (19), and have lower levels of PA for recreational and exercise purposes (20) and reduced access to PA facilities (21). Sustainable efforts to ameliorate health disparities must be multifaceted, including improvements of the built environment to be more supportive of PA (22). Expanding the availability and use of public transit–an essential component of the built environment–can play a significant role in increasing opportunities for PA, thereby creating an environment that supports healthier weight and overall health and well-being (23–26).

### 1.2. Public transit use as a public health approach

Access to public transit is associated with increased PA through transit use itself (e.g., walking to/from stations) and increased use of PA resources (e.g., parks and trails) (27, 28). Empirical evidence suggests that public transit is positively associated with walking and PA (29–31). A recent systematic review on light rail transit and PA, published in 2022, reported that all reviewed studies including six natural experiments found positive relationships between light rail transit and walking (32). Existing studies exploring the impact of public transit on PA focus primarily on light rail transit. Limited research has investigated BRT and its impact on PA. We only found one study carried out in Mexico City, which suggested that BRT and the implementation of the “complete street” strategies positively impacted transportation walking among catchment area residents (33). This study further indicated the varying impacts of BRT on subgroups with different sociodemographic backgrounds, demonstrating that women with low levels of education had the greatest increase in transportation walking (33).

There have been few studies examining transit-PA relationships through a natural-experiment design. Brown and Werner (34) used a natural experiment to explore the impacts of a newly opened stop along the TRAX light rail line in Salt Lake City, Utah on residents’ behaviors and attitudes. Hong et al. (35) examined the opening of a light rail corridor on residents’ travel and PA in Los Angeles, California. A recent natural-experiment study examined the causal effects of a new metro system on travel behavior in Nanchang, Jiangxi province, China (36). Choe et al. (37) investigated the trade-off effects of a new metro line in Hong Kong on transit and non-transit related PA among older adults, which suggested an increase in transit-related PA while a decrease in non-transit related PA. However, to the best of our knowledge, no studies have investigated the impacts of BRT, an increasingly popular form of public transportation, on PA among vulnerable populations using a natural-experiment study approach.

BRT systems are bus transit systems that typically feature a limited number of well-designed stations, traffic signal priority, and dedicated lanes. They combine the capacity, speed, and reliability of rail transit with the flexibility and cost advantage of a conventional bus system (38, 39). The BRT lines in El Paso have a distinctive branding scheme, iconic stations, and signal prioritization that lengthens green traffic signals and reduces delay. Its vehicles, however, operate in mixed traffic.

Previous research shows that BRT systems can help relieve traffic congestion, reduce emissions, and increase mobility options for low-income populations; and they are especially suited for low-density cities in the US because they can be easily integrated with existing and future land use patterns, encourage intermodal connectivity, and induce transit-oriented development (TOD) (38, 40). Despite its strong potential as a PA-promoting, lifestyle-oriented, and sustainable urban mobility option, causal impacts of BRT on PA have not been examined but are needed to complement and expand existing evidence on the transit-PA relationship with light rail (26, 34, 35).

### 1.3. Underuse of public transit

Scholars and policy makers who advocate for public transit have to grapple with the fact that public transit makes up a very low mode share–4.17% among total commuters and 5.57% among Latino/Hispanic commuters, compared to 81.83% and 83.28% for private vehicles, respectively.^1^ A fundamental question is whether more people will use public transit. There is a dearth of information on transit-oriented multi-level intervention (TOMI) strategies to reduce multiple modifiable barriers to transit use simultaneously, including (a) limited or no access (e.g., no bus stops close to home and/or destinations), (b) poor service (e.g., infrequent and unreliable service), (c) poor bus/shelter conditions (e.g., lacking safety and comfort), (d) expensive transit fare cost, and (e) difficulty in using the bus or planning the route (41, 42). The fact that the majority of those with access to transit (operationally defined as living in a 0.5-mile transit catchment area) still do not use public transit presents a need to address these other common barriers to promote transit use. A fundamental research gap is how improved transit access impacts transit use among underserved populations. Another important gap is whether specific TOMI strategies aimed at addressing common transit barriers might promote transit use, and subsequently improve PA and related health outcomes.

### 1.4. Our natural-experiment study

To address some of these important, unanswered questions, our transdisciplinary team of scholars from public health, urban planning, transportation, landscape architecture, and community engagement has carried out a natural-experiment study (2015–2022) to evaluate the impacts of a new major public transit system on PA and examine the effects of TOMI strategies. Sun Metro in El Paso, Texas opened two connected BRT lines in September 2019, which served many neighborhoods with high obesity rates, majority Latino/Hispanic residents, and low socioeconomic status. We gathered data from sensors (accelerometers and GPS), GIS (Geographic Information Systems) applications, and various instruments such as surveys, travel logs, and interviews. In addition to evaluating the environmental intervention–the opening of new BRT lines–we carried out three TOMI experiments referred to as–Bus Buddy, Free Fare, and First/Last Mile–to address knowledge, financial, and access barriers to transit use, respectively.

This paper aims to introduce our study protocols using a natural-experiment approach and share insights on how our transdisciplinary team overcame challenges faced during the seven-year project period, including delays in the BRT opening (the main intervention), the COVID-19 pandemic, methodological challenges, participant recruitment and retention, and predatory survey takers.

In concert with public health officials’ and policymakers’ desires to increase PA at the population level, this study targets a community-level intervention (i.e., transit service) holding the strong potential to encourage an active lifestyle of its members, especially those with limited mobility options and at high risk of physical inactivity. We target BRT, which has many unique advantages as described earlier but has not been examined in a natural-experiment study to the best of our knowledge. We also employ a rigorous research design that includes two comparison groups (non-exposed participants who live outside the BRT catchment area and non-users who do not use BRT), going beyond the typical design that involves the pre-post assessments of the cases only in most previous studies. Also recognizing the difficulties in reliably assessing the PA impacts resulting from BRT use, especially in natural experiments where various extraneous factors play a role, we use previously validated and objective methods to assess PA.

## 2. Methods and protocols

### 2.1. Proposed specific aims and hypotheses

We investigate the PA impacts of BRT, by (a) conceptualizing multi-level factors influencing transit use, (b) including multiple comparisons, and (c) applying promising methodological approaches used in other fields, for more contextual understanding of transit-PA relationships. The followings are our three aims and their associated hypotheses (for Aims 1 and 2) and research questions (for Aim 3).

#### 2.1.1. AIM 1-primary: determine PA impacts of BRT

*H1A*: Compared to the *pre-opening* baseline (Wave 1) and the non-users, the BRT users will have increased PA in both the short-term (Wave 2) and the long-term (Wave 3) *after* the BRT opening.

*H1B*: Compared to the *pre-opening* baseline and non-exposed participants, the BRT-exposed participants will have increased PA in both the short-term and the long-term *after* the BRT opening.

We define users as those who use BRT at least once per week. Non-users are those who do not use BRT on a weekly basis. We define exposed participants as those who live within 0.5 mile of a BRT station. Transportation planning studies have reported that employment, population, and renters within 0.5 mile of transit stations were significantly associated with transit ridership, and considered 0.5 mile as a typical threshold to define catchment areas (43–46). To account for potential local variations, we used the 1 mile threshold for the non-exposure participants to minimize/eliminate the potential BRT exposure effect in the 0.5–1 mile buffer area.

#### 2.1.2. AIM 2-secondary: examine PA impacts of additional TOMI strategies

Immediately after Wave 2, exposed but non-user participants (*n* = ~450) are randomly assigned to one of the three sub-groups (*n* = ~150 each): Bus Buddy, Free Fare, and First/Last Mile. Compared to those not receiving additional TOMI and to their own short-term (Wave 2) measures:

*H2A*: (Bus Buddy) Those receiving a one-time personalized training will increase BRT use and PA in the immediate follow-up assessment.

*H2B*: (Free Fare) Those receiving an introductory free weekly pass will increase BRT use and PA during the intervention exposure period (i.e., while the free pass is active).

*H2C*: (First/Last Mile) Those who get free Uber rides to BRT stations will increase BRT use and PA during the intervention exposure period (i.e., while the Uber rides are actively provided/scheduled by the team).

#### 2.1.3. AIM 3-tertiary: explore the benefits and costs of BRT implementation, and barriers and facilitators of BRT use

Using surveys, scenario-based ridership modeling, and a citizen science strategy, we explore two sets of exploratory research questions.

*Q3A*: Will the BRT implementation lead to higher societal benefits (e.g., obesity-reduction health care cost savings, reduced congestion/emissions/crashes) relative to costs (e.g., construction, operation, and maintenance)?

*Q3B*: What are the common barriers and facilitators of BRT use, and which of those are modifiable?

### 2.2. Conceptual framework

**Figure 1:**
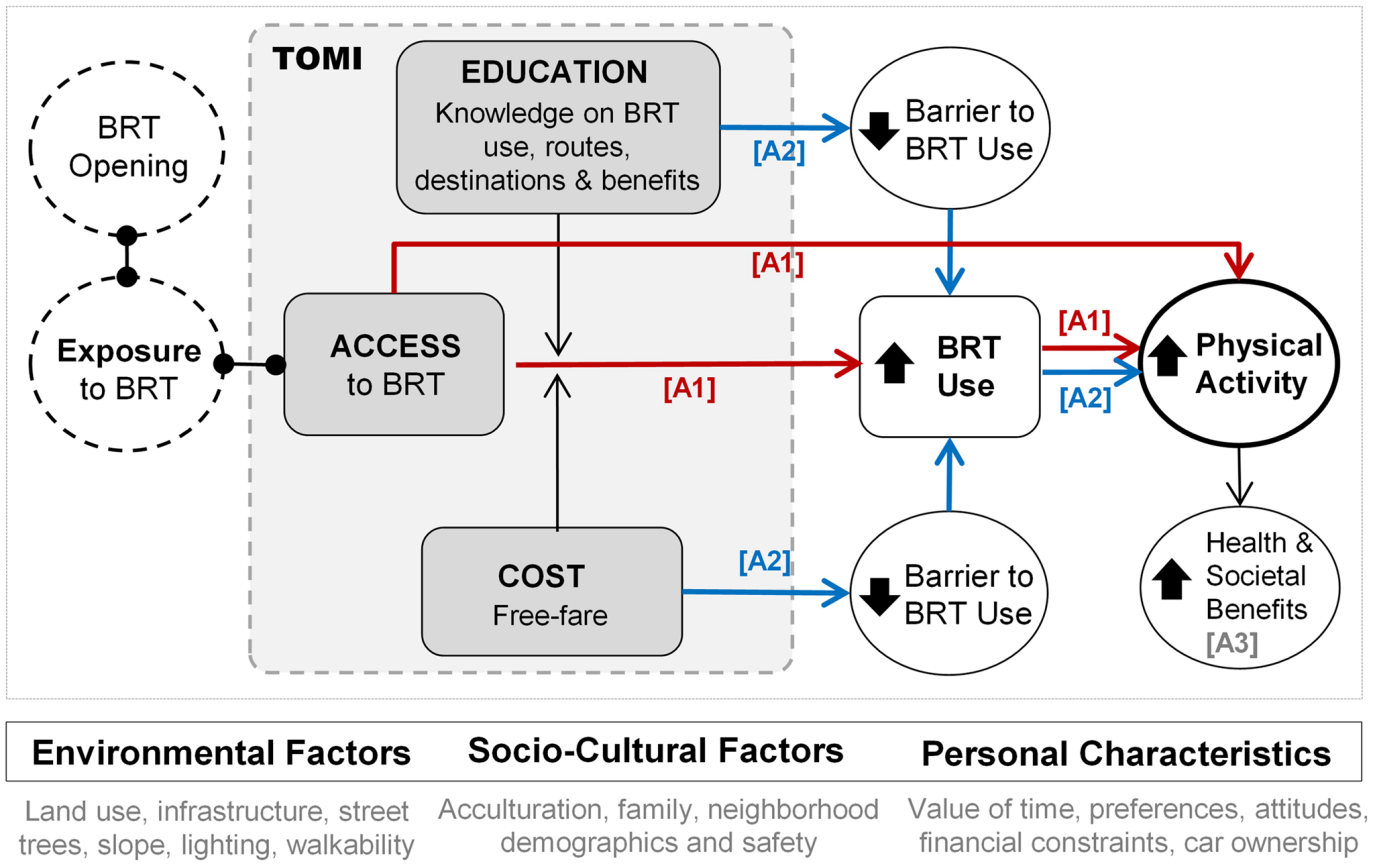
Conceptual framework. A1: Aim 1, A2: Aim 2, and A3: Aim 3.

We used the social ecological model as a broad conceptual foundation (47). The multilevel influences of behavior change and the reciprocal relationships between PA and external factors guided the conceptualization of this research. The selected TOMI strategies respond to the three major domains of PA determinants drawn from the social ecological framework: environmental, policy, and personal (Figure 1). The explicit consideration of both the use and the exposure benefits of BRT was motivated by the economic valuation literature on the use and non-use (exposure) values of an amenity, which is the BRT in this study (48). The direct use value of BRT is the increased PA resulting from walking/bicycling to/from transit stations. The indirect (non-use) value is expected from being exposed to the PA-promoting conditions that often accompany transit services such as increased safety and comfort (e.g., bus shelter with benches, lighting, and people), infrastructure improvements (e.g., connected sidewalks and bike lanes), and destination land uses (e.g., commercial and service uses that new transit stations often generate). Thus, in terms of the benefits for those not using the BRT, the TOD associated with the expected increased population movement in and around the intervention areas would generate new businesses and other amenities that may benefit those in the surrounding areas, regardless of whether they use the bus or not. For BRT users, this expected benefit from TOD would also be present, but with the additional benefit from the expected increased PA associated with trip-based PA. Replacement trips and induced trips are also important considerations in this study. In addition to the hypothesis that BRT encourages people to replace existing automobile trips, we also tested the hypothesis that BRT will stimulate new trips to destinations including parks, community centers, and trails along the new corridor that can further contribute to increasing PA.

### 2.3. Study setting and population

**Figure 2:**
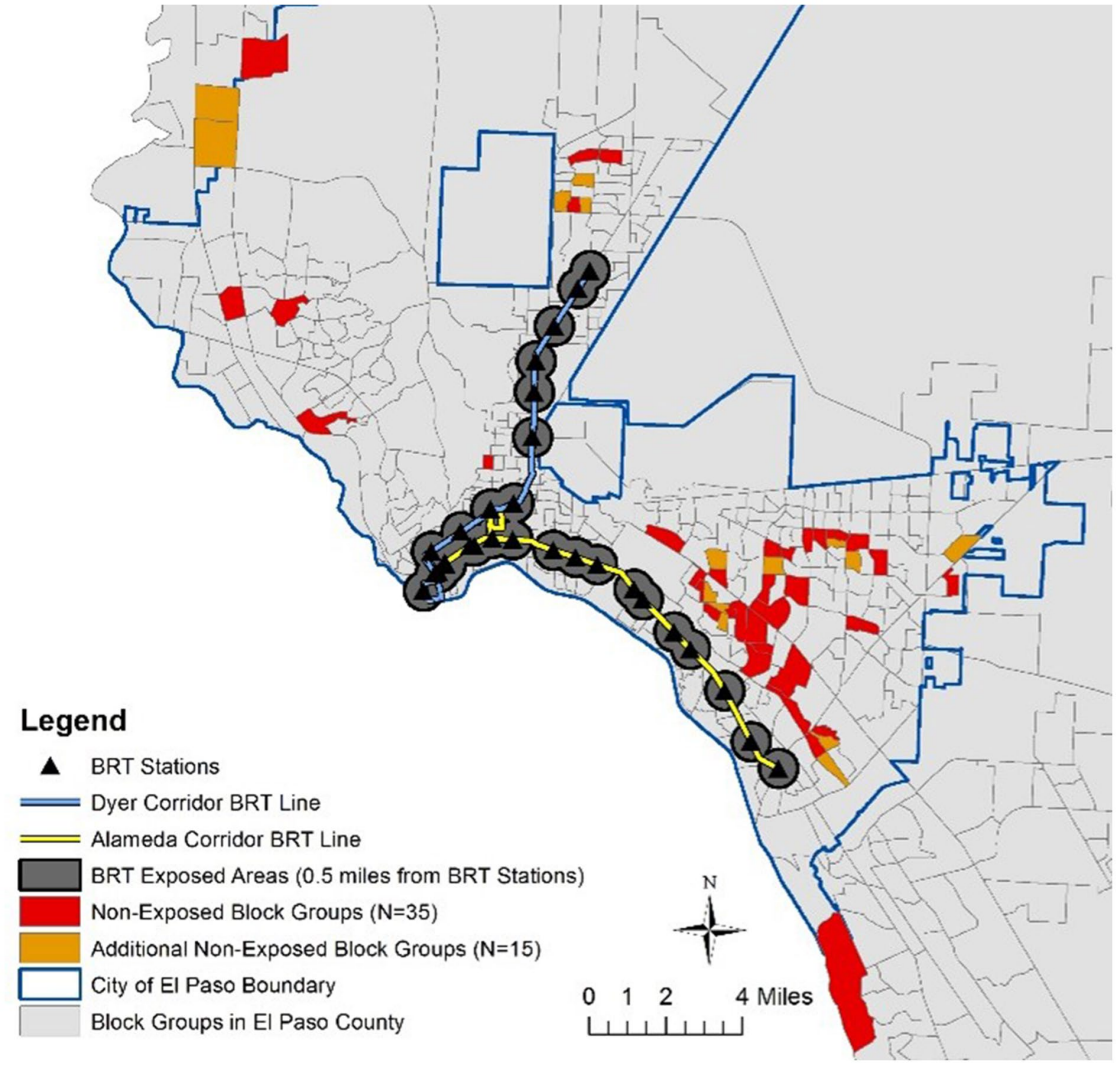
Study setting. The additional 15 non-exposed block groups were backup areas that were not used in this study.

In Fall 2019 (originally planned for Fall 2018), the City of El Paso, Texas opened two BRT corridors–Alameda Avenue and Dyer Street, which are connected at the Downtown Transfer Center (see Figure 2). Several amenities were also added to BRT station areas, such as free Wi-Fi, translucent panels for better lighting, bike racks, shade screens, electronic real-time displays, and ticket vending machines. The areas around some of the new BRT stations experienced infrastructure improvements (e.g., sidewalks, crosswalks, signage/signals, lighting, bike lanes) and new housing, service, and retail developments.

Table 1 presents a summary of various physical environmental and socio-demographic characteristics along the two BRT corridors in comparison with the City of El Paso. Compared to the rest of the city, the areas close to the new BRT stations have a similar coverage of parks, open spaces, and pedestrian infrastructure; however, these areas have a higher density of population and destinations, and better street connectivity. While the age and racial/ethnic composition in the study areas are similar to the rest of the city, residents living in our catchment area on average have lower education attainment, income level, and car ownership.

**Table 1:** Physical environment and socio-demographic characteristics of the Alameda and Dyer corridors.

	Features	City of El Paso	Alameda Corridor		Dyer Corridor		Alameda + Dyer	
			0–0.5 miles	0–1.0 mile	0–0.5 miles	0–1.0 mile	0–0.5 miles	0–1.0 mile
Physical environments	Population density (persons/acre)	3.94	6.76	6.76	8.33	7.47	7.41	6.87
	Transit availability^a^ (miles/100acres; miles/1,000 persons)	0.42; 1.05	2.32; 3.25	1.59; 2.48	2.24; 2.52	1.52; 2.05	1.90; 2.43	1.24; 1.85
	WalkScore^b^	39.00	54.23	48.40	61.89	52.16	54.83	45.68
	Commercial land use (%)	12.07	23.70	15.58	24.70	14.58	21.64	13.48
	Street connectivity (intersections/100 acres)	13.31	27.85	23.21	31.97	27.75	28.20	23.72
	Sidewalk coverage^c^ (%)	62.42	67.16	66.21	59.16	58.35	63.77	62.17
	Parks and open space coverage (%)	1.99	3.06	5.32	1.35	1.85	2.45	4.17
	Other PA resources (fitness/community center, school, etc.) (number/1000acres)	2.15	5.70	4.80	6.11	4.75	5.70	4.60
Socio-demographic characteristics^d^	Hispanic or Latino (of any race) (%)	79.89	94.69	94.32	82.88	81.42	88.52	87.50
	White (%)	83.49	86.47	86.51	83.87	83.99	85.35	85.60
	Population under the age of 18 (%)	27.36	24.11	24.47	26.58	26.53	25.81	26.15
	Population at or above the age of 65 (%)	12.25	18.66	18.38	14.90	15.13	16.54	16.75
	Population with ≤high school education (%)	46.69	72.21	68.60	63.66	60.88	67.25	64.21
	Mean household income	$58,012	$32,043	$34,662	$32,946	$37,737	$33,475	$37,030
	Average median household income	$43,887	$25,167	$26,898	$28,285	$33,108	$27,923	$31,441
	Carless households^e^ (%)	8.59	22.77	21.23	24.65	20.44	21.31	18.24
	Commuting by transit (%)	2.05	8.67	6.46	7.94	6.34	7.00	5.28
	Population (number)	651,590	46,394	109,056	43,798	99,294	78,364	171,772
	Housing units (number)	233,499	18,011	41,397	17,517	38,689	30,274	64,169

All physical environment variables were measured using ArcGIS, and socio-demographic variables were based on 2015 ACS 5-year population estimate. ^a^The transit availability variable was measured based on total bus route length. ^b^The value of the WalkScore at the city level was provided from Walkscore.com, and WalkScore of Alameda and/or Dyer Corridor was calculated based on the average Walk Score for each centroid of each 800 ft. grid within the boundary of Alameda and/or Dyer Corridor. ^c^The sidewalk coverage variable was calculated by the following equation: 100 × Sidewalk miles/2 × Street miles. ^d^Socio-demographic statistics were measured based on the proportion of block group area within the City of El Paso or the ½ mile buffers of Alameda and/or Dyer stations. ^e^Carless households variable was based on 2015 ACS 5-year population estimate.

Based on the 2011 SMART BRFSS County data, 43.30% of the population in El Paso County did not meet the level of recommended PA (150 minutes of moderate or 75 minutes of vigorous or an equivalent combination each week, as 1 vigorous-intensity minute of PA would be equivalent to 2 moderate-intensity minutes) (49). Considering the total population in the study corridor, an estimated total of 31,300 individuals lived within a 0.5 mile of the new BRT stations and was our target study population from which BRT-exposed participants were recruited. According to the ridership count and survey data provided by Sun Metro, transit provider in El Paso, the estimated daily riders for the Alameda corridor was about 3,450, including 2,060 new transit users and 1,390 current transit riders. The estimated daily riders for the Dyer corridor was about 3,100, including 1,851 new transit users and 1,249 current transit riders. Therefore, we estimated that about 3,300–5,000 BRT users would live within 0.5 miles of the new BRT stations. Based on a transit riders’ survey by Sun Metro, 91% of transit users walked or biked to access the transit stations.

### 2.4. Defining case and comparison groups

According to our original plan, the case-comparison design of the study would involve 750 exposed (living within 0.5 miles from a new BRT station) and 500 non-exposed (living beyond one mile from any BRT stations) adult participants who live in the city and do not meet weekly PA recommendations (150+ minutes of moderate-to-vigorous PA or MVPA, or an equivalent combination of MVPA) at baseline. Among those exposed, two groups were defined, including users (riding BRT 1+ times/week) and non-users (0 times/week). We planned for three waves of data collection, at baseline (Wave 1) and two post-exposure time points (Wave 2 and Wave 3) including shortly after opening (Wave 2), and long term after opening (Wave 3). We used multiple case-comparison groups in this study to help untangle the causal impacts of BRT. Specific definitions of cases and comparisons for each hypothesis are explained below.

For Hypothesis 1A, both cases and comparisons were from the exposed population (i.e., those living within 0.5 miles from any new BRT stations) who did not meet the PA guideline at baseline (Wave 1). Cases (Users *N* = 250) are those who became BRT users shortly after the opening (Wave 2). Comparisons (Non-users *N* = 500) were those who do not use BRT at Wave 2. Previous research showed that intention and attitudes toward transit were strong predictors of transit use (50–53). For Hypothesis 1B, Cases (Exposed *N* = 750) were all those exposed participants eligible for Hypothesis 1A. Comparisons (Non-exposed *N* = 500) were those who did not meet the PA guideline and lived beyond one mile from any BRT stations. Based on our original plan, for Hypotheses 2A-2C, all exposed, non-users (*N* = 450 after 10% attrition) at Wave 2 would be randomly assigned to one of the three aforementioned TOMI sub-groups. These TOMI interventions would be examined for their immediate short-term impacts at Wave 2 follow-up.

In determining comparison areas to be targeted for the *exposed* vs. *non-exposed* comparison, we used the Propensity Score Matching (PSM) method to identify the census block groups that are the most comparable to the census block groups where case participants live. PSM, as validated by previous social epidemiology studies (54) can help examine the conditional probability of a participant receiving a treatment based on a set of observed covariates, and thereby, reduce the selection bias by equating groups based on these covariates (55–57). In this study, we matched 50 census block groups (total population: 78,364) located within 0.5 miles of the new BRT stations with 35 census block groups (total population: 53,630) at least one mile away from these new BRT stations by using the R PSM package (see Figure 2).

Our matching was based on population density, percentage of population under poverty line, percentage of carless households, percentage of Hispanic population, and percentage of population with education less than high school. The selection of these covariates was informed by previous studies (58–60). The validity of our causal inference is based on the three basic assumptions of Rubin’s causal framework as summarized in previous studies (61, 62): first, potential outcomes are independent of the treatment assignments (63); second, matched subjects have a non-zero probability of receiving the treatment (64); and third, a subject’s potential outcome is not related to the treatment status of other subjects (65).

### 2.5. Study variables

#### 2.5.1. Objective physical activity measurements

The PA outcome measure in this study was the total weekly minutes of PA by intensity (e.g., light, moderate, and vigorous intensity) captured from the accelerometer (see Table 2). We highlighted MVPA because of its comparability with other studies, including several large-scale national studies that have used accelerometer-measured MVPA as their target outcome (66), and for consistency with CDC-recommended PA guidelines. *Walking* is also addressed separately as it is the most common transit-related PA type and will be detected based on the accelerometer, GPS, and travel log data (67–69).

**Table 2:** Study variables and measurement instruments.

	Hypotheses/Aims	Variables	Measurement instruments
Outcome variables	1A; 1B; 2A; 2B	Total PA (objective measures): Weekly total minutes of PA by intensity, calculated as light (200–2,690 CPM), moderate (2691–6,166 CPM), and vigorous (6,167+ CPM) (70–72); Weekly total minutes of walking	Accelerometer: ActiGraph GT9X or GT3X+ GPS: Qstarz BT Q1000 XT Travel Log: Research Team’s own instrument (Appendix 3) GIS: ArcGIS by ESRI
		PA (subjective measures): Self-reported behaviors, including the number of days of light, moderate, and vigorous PA per week, the average number of minutes of PA per day, and the type of physical activity.	These will serve as general screening and descriptive items. Relevant items from “2013 BRFSS Questionnaire” (79) and “The International Physical Activity Questionnaire (IPAQ)” (111)
	2A; 2B; 2C	Use of BRT (weekly frequency)	Travel Log; GPS; Survey (Appendix 1)
	Aim 1	Use of PA resources (parks, trails, gyms, etc.) by BRT (weekly frequency)	GIS (distance, route); Travel Log (destinations, time); GPS (location, speed)
	3A; 3B	Socio-economic benefits of BRT implementation; Barriers and facilitators of BRT use	Accelerometer; Relevant items from “2013 BRFSS Questionnaire” (79) and “The International Physical Activity Questionnaire (IPAQ) (111); Citizen science and community health assessment approach (112); Use of secondary data sources to estimate medical and societal cost data based on PA use (40, 113)
Mediating variables	1A; 2A; 2B	Use of BRT (weekly frequency)	GPS: Qstarz BT Q1000 XT; Tracking *via* free bus passes
Intervention variables	1A; 1B	Having access to BRT or not (living within 0.5 miles of a new BRT station vs. beyond one mile from any BRT stations)	GIS: Distance calculation functions
	2A; 2B; 2C	3-category variable: Receiving education, cost, first/last mile	Survey: Use of smartphone app; Confidence in using transit; Knowledge about transit benefits and BRT-reachable PA resources; Use of the free one-month transit pass
Confounding variables	1A;1B; Aim 3	Environmental factors, including land use mix entropy (0–1), various walkability measures, and traffic speed and volume; Perceived environmental variables (e.g., barriers and facilitators of BRT use)	GIS: Spatial analysis functions including distances and buffers; Raw GIS data (e.g., land use, sidewalks) from the City of El Paso, Sun Metro, and El Paso Metropolitan Planning Organization; Walkability measures partially based on Street Smart Walk Scores from walkscore.com Survey: Pilot study survey developed by the research team
	1A; 1B; Aim 3	Personal characteristics, including perceived barriers and facilitators of BRT use, distance to and mode use to access BRT, value of time, attitudes toward public transit and PA, financial constraints, and automobile availability	Survey: Relevant items from “Healthy Community Survey” (80), which was adapted in part from the “Twin City Walking Study,” the “Active Where Survey,” the “Neighborhood Environment Walkability Scale (NEWS)” (81–83, 89), “2013 BRFSS Questionnaire” (79), and pilot study survey developed by the research team
	1A; 1B; Aim 3	Socio-cultural factors, including perceived barriers and facilitators of BRT use, family pressure, acculturation, neighborhood demographics, and neighborhood safety	Survey: Family pressure measured in the Transit Social Norms (50), acculturation measured as Acculturation Proxies (114); Neighborhood demographics measured using 2012–2016 American Community Survey 5-year estimates (to be released on December 7, 2017); Neighborhood safety measured objectively with traffic accident data and crime data from the City of El Paso, and subjectively with relevant items from “Neighborhood Environment Walkability Scale (NEWS)” (81–83, 89)

We recorded raw accelerometer data with a 30 Hz frequency rate, and converted them to 15 second epochs using an analog bandpass filter and ActiLife’s software. We calculated triaxial-based VM (vector magnitude) to validate wear time and categorize PA intensity levels. We chose the 7+ consecutive-minute length with a 2 minute tolerance length for the wear time validation purpose. A 90 minute window of consecutive zero counts and/or tolerance of 2 minute intervals of nonzero counts with up/downstream 30-min consecutive zero count windows are considered as non-wear time (70–72).

We describe our GPS and accelerometer data fusion work in the Supplementary material. A valid day is defined as having 10+ valid wear hours per day. Participants were asked to re-wear devices when they returned data of less than four valid days. Standard/accepted activity count thresholds (e.g., 200–2,690 counts/min for light, 2,691–6,166 for moderate, and 6,167+ for vigorous) are used to determine PA intensity levels (70–72). The GPS and accelerometer devices are set up to record the data at 15 second intervals and linked using the time stamp (69). We use a method previously developed by our research team to expedite the data integration, clean-up, and post-data processing (69).

#### 2.5.2. Subjective measurements of physical activity using surveys

Subjective measures of PA were captured using surveys. We used the International Physical Activity Questionnaire (IPAQ) (73) as a guide to develop survey items to capture light, moderate, and vigorous PA minutes per day and the number of days one typically engages in each level of PA intensity.

In addition to treating these separately for specific analyses of given intensity levels, we also combined moderate and vigorous intensity to calculate the MVPA minutes, following the guidance from other published studies (74, 75), to facilitate comparisons with national guidelines (76, 77) based on the weekly MVPA minutes.

#### 2.5.3. Socio-cultural and environmental variables

We generated these variables from surveys, including the intention to use transit, attitude toward transit and active transportation, socio-demographic (car ownership, acculturation, marital status, education, employment, household income), personal demographics (e.g., age, sex, race, health, and height and weight status for BMI calculations), commute travel mode, trip-making characteristics, residents’ value of time, neighborhood social-economic status, and perceived neighborhood characteristics such as crime, crash, traffic speed/volume.

We used ESRI GIS software (78) to perform various proximity and buffer analyses to generate objective measures of the neighborhood environment. Relevant measures cover land uses (e.g., percent of different land uses, residential density, distance to PA resources and other utilitarian destinations), street characteristics (e.g., street connectivity, sidewalk connectivity, crosswalks/intersections, posted speed), safety (e.g., crime and crash density, traffic speed), and natural environments (e.g., tree canopy coverage, greenery coverage, slope).

### 2.6. Data collection

We used surveys to capture all personal, socio-cultural, perceived environmental variables, and self-reported PA outcomes (e.g., PA by intensity and walking, BRT use, and use of PA resources via BRT). GIS including ESRI Business Analyst was used to objectively measure the environmental variables and several socio-cultural and neighborhood contextual variables derived from the census data. Accelerometer, GPS, and travel log captured the main PA outcome variables (weekly minutes of PA by intensity, and of walking) and other PA outcomes (use of BRT and use of PA resources by taking BRT). All data collection protocols and instruments were reviewed and approved by the institutional review board (IRB) of the institutions our study team members are affiliated with.

#### 2.6.1. Survey

A preliminary survey instrument was developed and tested during the pilot study. It required an average of 40 minutes to complete. The survey instrument was designed with validated and/or tested items from similar projects carried out by members of our study team, other relevant published surveys, and existing national survey questionnaires shown to have acceptable psychometric quality (79–87). The English version was developed first, and a forward-backward translation process (88) was used to develop the Spanish version to ensure the equivalence of the instruments in both languages.

The survey instruments included a screening section with questions on age, residential location, current PA level, difficulty in walking, and intention to use BRT to help recruit sufficient case participants for Hypothesis 1A. Intention was only one of the many indicators predicting behavior changes but had been shown to be highly correlated with the actual behavioral outcome (50). It also included control variables such as sex, race, ethnicity, education, marital status, household composition, car ownership, income, attitudes, social factors, and general self-assessed health (including number and type of co-morbid conditions). Perceived measures of dependent and independent variables were also captured from the survey, such as travel behavior and mode choice, facilitators and barriers to transit use, and environmental conditions around the home and BRT station areas.

#### 2.6.2. GIS and audit

Built (e.g., sidewalks, land uses, density), natural (e.g., tree canopy), and socio-behavioral (e.g., traffic, safety, transit ridership) environmental data were captured utilizing existing GIS data from the City of El Paso, Sun Metro, El Paso Metropolitan Planning Organization, and ESRI Business Analyst. We acquired most of the available GIS data during the pilot study. Incomplete or missing GIS data (e.g., crosswalks and streetlights) were manually captured through field audits and digitized for GIS analyses. Walkability measures also included Street Smart Walk Scores from walkscore.com.

#### 2.6.3. Accelerometer, GPS, and travel log

The primary outcome of PA was objectively measured using seven day data captured from the accelerometer (ActiGraph GT9X or GT3X+) (89). During the same seven days, GPS (Qstarz BT Q1000 XT) (82) and travel log data were collected to detect walking, identify the locations/purposes of PA, and isolate PA from traveling to/from BRT stations and by using PA resources by taking BRT (66, 90). We linked GPS data (e.g., time, location, speed) with the accelerometer data using the time stamp as the common link, in order to detect outdoor walking and other PA associated with BRT use (67, 68). Travel log data included origin and destination, start and end time, and trip purpose, providing additional data for cross-checking to increase accuracy and reduce errors in activity detection from devices (69). The research team developed a simplified travel log format which had been tested and used in similar projects (80).

#### 2.6.4. Methods for exploratory AIM 3–benefits and costs of BRT implementation (3A)

We used secondary data and previous case studies (91–94), as well as round table discussions and interviews for this aim. All available secondary data on BRT implementation costs and benefits were obtained from Sun Metro and relevant public agencies, and from published reports/documents. To explore BRT benefits, we carried out round table discussions and individual interviews with key stakeholders involved in the BRT implementation, such as public officials, developers, and special interest groups.

#### 2.6.5. Methods for exploratory AIM 3–barriers and facilitators of BRT use (3B)

We implemented a series of interviews/focus groups among diverse groups of participants to gain more in-depth information about the barriers and facilitators of BRT use. Building upon what we learned from the main study and the TOMI study, we identified key areas needing additional input/information from the participants. Accordingly, we developed the following qualitative studies (1): interviews among captive users, choice users, and nonusers (2); interviews of TOMI participants; and (3) interviews among stakeholders from local and regional government agencies and the development industry.

### 2.7. Data analyses

#### 2.7.1. Data preparation

We examined descriptive statistics and visual graphics to check the data distributions and identify potential thresholds for categorization, identify outliers and missing values, and check for any potential problems with the planned statistical methods. We also evaluated the population representativeness of the sample and checked the comparability between the case and comparison groups and the need for covariate adjustment. We assessed the patterns of missing data and conducted sensitivity analyses to evaluate the robustness of the methods used to treat missing data problems as they arose.

#### 2.7.2. Key analytical approaches

To strengthen the causal inferences from our natural-experiment design, we followed suggestions from previous studies (95–98) by making comparisons between different groups (e.g., users vs. non-users, exposed vs. non-exposed) and employing rigorous evaluation methods to address different sources of biases. Variables from surveys, GIS, and audits, mostly considered confounding factors, were considered in multivariate analyses. They were first evaluated for quality and distribution, and recoded as needed to ensure proper treatment in the statistical model. Given the large number of variables generated from these methods, data reduction strategies such as factor analyses and composite scoring methods were used to extract fewer variables representing domains of theoretically important factors.

For Hypothesis 1A, in addition to performing the commonly used *t*-tests among users and non-users and longitudinally, we used the Difference-in-Differences (DID) method to control for unobserved or imperfectly measured factors that influenced both BRT use and PA outcomes (96, 97). The DID method allowed us to compare pre-post intervention change in PA in the user group with that in the non-user group. We followed the conventional DID estimator’s assumption that the treated and control groups would have followed parallel trends over time (99). We used the *bootstrap* command in STATA to calculate pre-opening and post-opening means of PA for each group and the 95% confidence intervals; these calculations allowed us to estimate the change of PA in a treatment group relative to its control group. To further enhance the robustness in our DID estimations, we conducted sensitivity analysis using the *diff* module (100) in STATA; we added different combinations of socio-economic covariates to the DID analysis and test how these covariates influence the causal impact of BRT on PA. Following recent methodological developments, our team is currently exploring alternative approaches to jointly account for spatial spillovers, tackle confounding bias, and capture the time trend to evaluate the effectiveness of safety treatments (61).

For Hypothesis 1B, we used the PSM method to reduce the impact of confounding factors, by identifying non-exposed neighborhoods that shared similar socio-economic characteristics with exposed neighborhoods. Our matching was operationalized using the *psmatch2* command in STATA, guided by common matching schemes introduced by Guo and Fraser (62). Similar to the analysis in Hypothesis 1A, we performed *t*-tests among the exposure and non-exposure groups and longitudinally employed the DID method.

For Hypothesis 2A-2C, we performed paired-sample *t*-tests to compare the pre-TOMI and post/during-TOMI PA outcomes for each group. In addition, we had planned to employ the DID method to determine whether the three sub-groups treated by an additional TOMI had a higher PA increase than a control group that received no additional TOMI. However, we were unable to recruit the control group due to resource constraints.

For Question 3A, we used valid proxy measurement (e.g., housing/rental price, property value, business change) to quantify the economic impacts of BRT stations (e.g., walkability premium, new apartment development). We analyzed the changes in property values and in the number of jobs after the opening of BRT stations. Also, spatio-temporal distributions of the construction permit data were analyzed to explore the changes in the new or re-development activities along/near the BRT corridors. Using the smartphone mobility data collected by SafeGraph, we further conducted fixed effects models to explore the impacts of BRT corridor development on travel patterns (e.g., non-work-related activities) in El Paso, Texas.

For Question 3B, we utilized the 4 Ps of social marketing to guide the qualitative study data analysis process (1): Product–bus use (2); Price–barriers to bus use (3); Place–places visited using busses, accessibility, etc.; and (4) Promotion–facilitators to bus use. These social marketing strategies were applied to investigate modifiable environmental solutions for promoting bus use among different types of bus users (e.g., choice users, captive users). For the TOMI interviews, we used the Integrative Model of Behavioral Prediction to understand participant attitudes and beliefs regarding transit after participating in TOMI. Additionally, we also asked participants questions about their experience with TOMI and ideas they propose to improve the intervention or transit in El Paso.

#### 2.7.3. Power analysis

For Hypothesis 1A (users vs. non-users all of whom are exposed). Based on objectively measured PA, previous researchers found an effect size of 0.51 (87) for PA between residents exposed to different neighborhood walkability levels and 0.37 (101) for PA between transit commuters and non-transit commuters. Our pilot study found an effect size of 0.66 for weekly minutes of walking for transportation between exposed users and exposed non-users. Guided by these findings, we conservatively chose an effect size of 0.36. In a two-wave ideal pre-post sampling scenario, the subsample sizes of exposed users $n_{\exp\_user}=121$ and exposed non-users $n_{\exp\_nonuser}=121$ are required to detect the difference of the effect size $d_{1A}=0.36$ at the significance level of 0.05 with power 0.80. Therefore, $n_{\exp}$ =242 is needed to test Hypothesis 1A. In our sampling framework, we need to factor in the neighborhood clustering effects and attrition. We anticipated a small neighborhood intraclass correlation. Let *ICC* stand for the intraclass correlation coefficient, then the design effect is D=1+m∗ICC , where *m* is the average number of participants per neighborhood. We defined the neighborhood as the census block group and restrict *m* to be no more than 15. El Paso, as many other American cities, features homogenous auto-oriented development patterns. We assumed ICC = 0.02. Then our design effect was D=1+15∗0.02=1.30 . Based on previous evidence (102) and the consideration of some participants switching between the user and non-user groups, we expected a 10% attrition rate between Wave 1 and Wave 2, and 25% between Wave 2 and Wave 3. Accordingly, we expected 468 exposed residents at the baseline to test Hypothesis 1A.

For Hypothesis 1B (exposed vs. non-exposed). Assuming the same conservative effect size, neighborhood clustering effect and attribution rates as above, 468 residents exposed to BRT and 468 not exposed to BRT at baseline would allow us to gain a statistical power larger than 0.95.

For Hypothesis 2A-2C. Previous evidence suggested that free-fare policies could increase bus ridership by 56% (42). Assuming a conservative effect size of 0.40 for 2A and 2B, our relevant sampling plan (150 exposed non-users for each of the three TOMI-treated subgroups) would allow us to achieve a higher than 0.80 statistical power at the significance level of 0.05 for the TOMI assessment.

Based on the above analyses, we set our overall sampling goal in Wave 1 (baseline) as 1,250 participants: 250 BRT users and 500 BRT non-users from the 50 selected block groups within ½ mile of a BRT station, and 500 (non-exposed) participants from the 35 selected block groups beyond one mile of any BRT station.

#### 2.7.4. Strategies to reduce biases

To handle self-selection biases, we targeted participants living in the study community prior to the announcement of the BRT project groundbreaking in December 2016. We directly asked the participants about their preferences and attitudes that can lead to self-selection biases (e.g., residential preferences, reasons for choosing one’s household and neighborhood, attitudes and preferences related to PA) at multiple time points for both case and comparison participants. We also used statistical strategies, introducing a binary variable to denote residential choice in the model, as commonly used for treating self-selection problems in the sample selection (103) and modeling PA conditional on the model for residential choice that reflects self-selection.

## 3. Protocol implementation: challenges and experiences

When implementing the above protocols, we encountered numerous challenges, most of which were inherent to this type of natural-experiment study. The key to responding to these challenges is to document and monitor the study context and develop reasonable response strategies. We summarize the key challenges and experiences as follows.

### 3.1. Delays in the transit corridor opening

The rollout of the BRT, locally known by the name Brio, lines (i.e., the case intervention) within the case neighborhoods in the study area was delayed for more than 1 year due to various engineering, construction, and community factors. While diligently following up with Sun Metro, the local public transit operator in El Paso, to stay updated on the opening date, we took it as an opportunity to boost our baseline participant recruitment. However, the prolonged baseline data period not only meant we had to consider seasonal factors and monitor varying timelines of recruitment among hundreds of participants but also brought cascading delays to future waves of recruitment work. After we had just settled into the Wave 2 recruitment with a few hundred participants already recruited, the team was forced to stop recruitment abruptly due to the COVID-19 pandemic.

### 3.2. COVID-19 pandemic

The COVID-19 pandemic started in the US shortly after the BRT opening. The pandemic significantly delayed and compromised the integrity of the pre-post evaluations and the ability to safely recruit sufficient case and comparison participants of matching characteristics. Even in light of a relatively stronger study design of having a pre/post case–control design, the socio-economic environment (e.g., social interactions, business closures, alternative work arrangements, bus routes and timing) changed significantly, making it impossible to single out the impact of the BRT opening on PA and walking. Therefore, we decided to halt the Wave 2 data collection activities, and initiated several new activities while reprioritizing planned/proposed activities with the aim to still fulfill our primary aim from different angles.

These new and reprioritized initiatives included (a) designing a series of qualitative studies (e.g., interviews) addressing some of the compromised aims, (b) utilizing the secondary data (e.g., SafeGraph) to address BRT impacts using various existing proxy measures, and (c) employing spatial-analytical strategies to design case-comparison matching scenarios. To capture the potential built environmental changes that can impact BRT use, we also gathered the building permit data from the City of El Paso, and sorted out those that could impact the pedestrian and BRT use behaviors such as infrastructure constructions/improvements, major property developments, large-scale maintenance projects, and new pedestrian and bicycle amenities. Based on the timeline and locations of these activities, we identified those that can impact the BRT use and considered those as confounding variables during the data analysis process.

We also conducted pandemic-related surveys to better understand the impacts of COVID-19 on people’s daily travel and other activities (104). In some ways, this major change in the study protocol led to new research activities that served an important public health objective, thus responding to the ongoing and timely needs of advancing knowledge needed to protect vulnerable, underserved populations during a pandemic like COVID-19. This innovative and timely response to the public health crisis was made possible by the nature of our transdisciplinary team which covers public health, urban planning, landscape architecture, and transportation planning, with all necessary expertise to pursue such new/additional research directions effectively and efficiently.

### 3.3. Methodological challenges

#### 3.3.1. Timing

While multiple pre-post assessments were desirable, real-time execution of such strategies in our large-sample population study was challenging. Particularly, we found it difficult to determine the optimal timeframe for carrying out data collection during the pre- and post- intervention periods. It was unclear or unknown how long it would take for the intervention to show measurable effects and what was considered short-term vs. long-term impacts. Empirical or theoretical evidence was lacking to guide our selection of optimal time points. We referred to Supplementary data such as the ridership trend for new BRT lines elsewhere to guide our decision, but recruitment difficulties required us to expand beyond the initially determined timelines.

#### 3.3.2. Data

Due to the inability to fully control for the intervention and samples, issues such as sample biases, self-selection biases, and measurement errors, which were prevalent in previous natural-experiment studies, would likely pose a data quality threat to our study. As mentioned in previous studies, by combining self-report surveys with objective measures (e.g., accelerometer and GPS), we observed time lapses between the two types of measures. Our objective measures required participants to wear the devices for a full week for each wave of data collection. Some commented about the discomfort and privacy concerns of these devices. Many found it difficult to follow all the required protocols. Even with an in-person meeting to explain the protocols in detail and reminders for the study activities, about one third of the participants did not adhere to all study protocols, resulting in incomplete data to varying extents. Further pragmatic efforts such as frequent reminders and individually tailored assistance were added. We found that timely follow-up activities with participants would help participants to ease discomfort, build trust, and, as a result, better follow the study protocol. When designing the study protocol, researchers may consider a balance between participant burden and scientific rigor, which is key in these types of pragmatic efforts. Another important and related issue was data integration from different resources, including self-report (e.g., traditional surveys, travel log, interview), devices (e.g., accelerometer and GPS), and environmental data (e.g., GIS data, street audit data). We dealt with the data integration challenge by carefully studying the data structure and training our staff to use various open-source tools that were specialized in analyzing temporal and spatial data.

### 3.4. Recruitment and retention of participants

We encountered several recruitment challenges, including low-response rates of the target population, language barriers, verification issues with mail addresses, timing of delivering incentives, and no-shows for scheduled appointments. Several studies reported similar difficulties in recruiting participants of minority race and/or ethnicity in research (105, 106). Our recruitment success was based on a strong partnership with various community stakeholders. Since the early stages of the project, we worked closely with local stakeholders, bilingual researchers, project managers, and staff, who were sensitive to cultural norms in our study community. During the study design, they provided valuable feedback on the wording and translation of survey questions, structure of study incentives, local media channels, and locations of recruitment booths. For example, our team tested the survey wording in meetings with these community stakeholders, to ensure that the survey wording was not overly complicated, free of jargon, and likely to be understood. Multiple local stakeholders reviewed our professionally translated Spanish survey and other study materials, to ensure accuracy. The trusting relationships were further strengthened in the following project phases.

Recognizing the uniqueness of our study community being primarily Latino/Hispanic and located along the US-Mexico border, we tested 12 different recruitment strategies during the pilot phase. The USPS Every Door Direct Mail service, which allowed researchers to select spatially targeted delivery routes, turned out to be the most productive strategy and helped to yield about two thirds of the baseline study participants. Recruitment booths at public locations, as recommended by local collaborators, were proven to be a culturally sensitive strategy and helped to yield about one third of the study participants. Conventional approaches popularly used for large-scale population studies such as mass media, advertisement, and flyer distributions/postings were not effective in our study community. Despite the increased access to the internet and smartphones, we noticed a significant digital divide issue in our study areas. Distribution of recruitment information, therefore, could not be limited to only digital channels. We made a series of adjustments throughout the project to respond to evolving situations such as COVID-19 and staff/personnel changes. We discussed the challenges in our regular meetings and implemented strategies accordingly.

Our local staff were *promotoras*, a Spanish term for Community Health Workers. They played a pivotal role in implementing our culturally sensitive recruitment and management approaches (107). They were the first to point out the above digital-divide issue. We benefited from their suggestions to recruit participants in a face-to-face fashion. In efforts to boost retention, they continually checked in with participants regarding the progress during the study. Staff resources were allocated for meeting participants at their preferred locations, following up through calls, and managing rides for the TOMI intervention. We also held frequent team meetings to manage study incentives and logistics. Finally, we developed computerized data tracking and reminder systems with email-enabled notifications, to bolster our follow-up work.

Longitudinal studies need to diligently implement retention strategies to reduce attrition and ensure sufficient statistical power for longitudinal assessments. We found it useful to maintain frequent communication with participants and distribute study incentives in a timely manner. These actions require the team to pay attention to details and diligently monitor concerns raised by participants. In addition, we found open house events to be useful for participant recruitment and engagement. Prior to COVID-19, such events allowed us to connect with participants, who met the research team in person; participated in fun, engaging educational activities; and received project-branded tokens of appreciation (e.g., water bottles with study logo). Such events helped us build trust with participants, and more broadly, with the community.

### 3.5. Deterring predatory survey takers

During the baseline recruitment work, we had to deter numerous predatory survey takers. We defined them as those who used bots to sift through online platforms, such as Qualtrics, SurveyMonkey, LimeSurvey, and Google Form, for surveys that would provide monetary incentives (108). They would then automatically and abusively complete surveys to claim the incentives. In addition to financial loss, researchers would risk their study results being contaminated by the numerous invalid responses. After detecting them, our team implemented the following measures (1): creating new survey links (2), adding a spatial screening process to screen out fake or ineligible home addresses (3), adding Captcha authentication (4), adding a warning message to the information page (5), limiting to only one IP address per survey, and (6) limiting the gift card delivery to a home mailing option only (removing the e-Gift card option). These measures, along with the daily monitoring of the survey data, successfully prevented further attacks.

## 4. Conclusion

This paper addresses a major research gap in public health: How public transit infrastructure can serve as an instrument to promote healthy aging by encouraging physical activity? And accordingly, how to use the natural-experiment approach to reveal the transit-health causal relationship. This paper contributes to filling this gap by introducing our seven year natural-experimental study’s original protocols related to research rationale, hypotheses, design, and measurement and analytical approaches. Our protocols describe our natural-experiment study, which was carefully designed around our conceptual model and an innovative case–control design that involves participants exposed/non-exposed to new BRT stations. We acknowledge multiple challenges to implementing the protocol, and strategies employed to turn challenges into opportunities for new research. We have made a case for why a natural-experiment approach allows us to understand the lifestyle impacts of increased access to public transit. The causal effects identified by such an approach serve as evidence to reform policies that lead to improved health. We experimented with some potential policies–TOMI intervention strategies–to examine how these strategies, by impacting transit use, may be linked to improved PA and overall community health outcomes.

Our transdisciplinary approach was pivotal to how we persevered through seven years of the project period. We utilized a team approach that included individuals with expertise in urban planning, landscape architecture, public health (health behavior, public policy, health inequities), geospatial analyses, and relevant local experience. Our team included researchers, Community Health Workers, and local leaders, along with partnerships with the public sector. This approach was not without challenges, given different disciplinary perspectives, terminology, and training in statistical methods and reporting standards. Our team members included those with valuable experience with participant recruitment in this unique population and qualitative research methodology, as well as large-sample survey data collection integrated into GIS. The diversity of experience and the complementary nature of the team’s expertise was valuable. This approach was transdisciplinary (109) due to, among other things, using a conceptual framework that cut across multiple fields represented in the project. Given this insight, we recommend other studies consider the feasibility and potential value gained in taking a transdisciplinary approach or other approaches that cut across disciplines (e.g., multidisciplinary, or interdisciplinary research) (110), rather than deferring to what might be considered a more typical siloed approach, where feasible for a given project.

The following are our key takeaways for future researchers. First, do not be stubborn, have Plan B. We shall always have a lower-risk backup plan. The COVID-19 pandemic provided many unanticipated challenges, namely a pause in research activities, recruitment challenges, and concerns about data interpretations due to many confounding factors. When faced with the pandemic that impacted nearly every aspect of daily life for many, including public transportation travel patterns, our team made a lower-risk backup plan, which guided us to assess the situations diligently and adjust our study strategies quickly to minimize the impact on the scientific merits we originally proposed.

Second, do not be afraid, view challenges as new opportunities. Natural-experiment researchers shall always be aware of, and receptive to, new research directions, even those that may have not naturally emanated from our originally planned efforts at the proposal stage. Even though our Wave 2 data collection was interrupted by the COVID-19 pandemic, we were able to carry out multiple layers of subjective and objective measurements, revealing the roles of transportation infrastructure in promoting public health. We experimented with three different TOMI strategies to facilitate bus ridership in a predominantly low-resource community. Given that ridership declined during the pandemic, findings about the benefit of different TOMI strategies for increasing ridership will be extremely valuable. Further, our ongoing qualitative interviews with key stakeholders will provide insights into the role of transit in promoting the health and economic well-being of underserved populations across the life-course.

Third, do not be shy, build trust and relationships with study participants and local community stakeholders. Our project involves recruitment across multiple years, meeting with participants and asking them to allow us to measure their daily activities (e.g., MVPA, GPS movement). All these tasks require us to carry out active and diligent engagement with local community stakeholders and study participants, *via* community events, local recruitment booths, phone calls, and digital engagement tools such emails and texts. When people realize that you do research “with” them, instead of “on” them, they would trust you, follow protocols, and spread the words for you.

Future researchers, practitioners, and policy makers who are interested in producing and consuming natural-experiment research need to acknowledge the importance of being flexible and ready to adapt to new circumstances. They need to be versatile and make swift adaptations in a world characterized by volatility and uncertainty.

## Data availability statement

The raw data supporting the conclusions of this article will be made available by the authors, without undue reservation.

## Ethics statement

The studies involving human participants were reviewed and approved by Human Research Protection Program, Texas A&M University. The patients/participants provided their written informed consent to participate in this study.

## Author contributions

WL, CL, MO, SZ, ST, MX, XZ, SL, SW, RA, EG, and LW contributed to the original proposal, which was funded by the National Institutes of Health. WL, CL, MO, and XZ contributed to the paper’s conceptualization. WL, CL, MO, SZ, ST, and XZ contributed to writing the first draft and making significant editing. All authors contributed to reviewing and proofreading the paper.

## Funding

This study was funded by the National Institutes of Health (Award#: 1R01CA228921–01).

## Conflict of interest

The authors declare that the research was conducted in the absence of any commercial or financial relationships that could be construed as a potential conflict of interest.

## Publisher’s note

All claims expressed in this article are solely those of the authors and do not necessarily represent those of their affiliated organizations, or those of the publisher, the editors and the reviewers. Any product that may be evaluated in this article, or claim that may be made by its manufacturer, is not guaranteed or endorsed by the publisher.

## Footnotes

^1^We calculated the percentages based on the United States Census Bureau 2021 ACS 5-Year Estimate Subject Tables S0802: Means of Transportation to Work by Selected Characteristics.
