# Supplementary material for: Examining the impacts of public transit on healthy aging through a natural experiment: study protocols and lessons learned from the Active El Paso project
Source: Front Public Health. 2023 Jul 25;11:1132190. doi: 10.3389/fpubh.2023.1132190 (PMC10415912; doi:10.3389/fpubh.2023.1132190)
Supplement: Supplementary file 1 [file Data_Sheet_1.docx]

***Supplementary Material:***

***GPS and Accelerometer Data Fusion Work***

**Manuscript Title:**

**Examining the Impacts of Public Transit on Health through a Natural Experiment: Study Protocols and Lessons Learned from the Active El Paso Project**

**Wei Li^1,2,3*†^, Chanam Lee^1,2†^, Sinan Zhong^1,2^, Minjie Xu^1,2^, Samuel D. Towne Jr. ^4,5,6,7,8^, Xuemei Zhu^2,9^, Sungmin Lee^1,2^, Suojin Wang^10^, Rafael Aldrete^1,1^, Eufemia B Garcia^1,2^, Leah Whigham^13^, Ashley M. Toney^13^, Jorge Ibarra^1,2^, Marcia G. Ory^4,8†^**

^1^Department of Landscape Architecture and Urban Planning, School of Architecture, Texas A&M University, College Station, Texas, USA

^2^Center for Health Systems and Design, Texas A&M University, College Station, Texas, USA

^3^Center for Housing and Urban Development, Texas A&M University, College Station, Texas, USA

^4^Department of Environmental and Occupational Health, School of Public Health, Texas A&M University, College Station, Texas, USA

^5^School of Global Health Management and Informatics, University of Central Florida, Orlando, Florida, USA

^6^Disability, Aging, and Technology Cluster, University of Central Florida, Orlando, Floria, USA

^7^Southwest Rural Health Research Center, Texas A&M University, College Station, Texas, USA

^8^Center for Community Health and Aging, Texas A&M University, College Station, Texas, USA

^9^Department of Architecture, School of Architecture, Texas A&M University, College Station, Texas, USA

^10^Department of Statistics, College of Arts and Sciences, Texas A&M University, College Station, Texas, USA

^11^Texas A&M Transportation Institute – El Paso Office, El Paso, Texas, USA

^12^Colonias Program, School of Architecture, Texas A&M University, Texas, USA

^13^Center for Community Health Impact and Department of Health Promotion and Behavioral Sciences, School of Public Health, The University of Texas Health Science Center at Houston, El Paso, Texas, USA

*** Correspondence:**Wei Li
[wli@tamu.edu](mailto:wli@tamu.edu)

†These authors contributed equally to this work and share first authorship.

Global Position System (GPS) and accelerometer units are becoming increasingly popular in built environment-PA research in recent years. Especially for those who concern about the impacts from the built environment on people’s health-related behaviors, these tools provide an unprecedented opportunity to objectively measure physical activity intensity for all daily routines and the geographic locations for all outdoor behaviors. They could also be used for validating the conventional self-report data collection methods.

Pilot studies have been conducted by our team to explore the strengths and limitations by employing GPS and accelerometer. Undoubtedly, with the concurrent measurement of activity levels as well as the locational coordinates, we can visualize different activity intensity over time and space (Table S1 & Figure S1). Furthermore, synthesizing the data from both devices allows for detecting travel modes and linking with the environmental settings (Figure S2).

Table S1: Accelerometer counts and activity level for adults*

| Activity Level | Activity Count Corresponding to Level |
| --- | --- |
| Sedentary | 100 or less counts (preliminary) |
| Light | Above sedentary to 1952 counts (less than 2.99 METS) |
| Moderate | 1953 to 5724 counts (3.0 to 5.99 METS) |
| Vigorous | 5725 to 9498 counts (6.0 to 8.99 METS) |
| Very Hard | Greater than 9498 counts (greater than 9.0 METS) |

* activity count thresholds differ slightly from what we propose to use in the proposal

as recommended by newly available evidence since the pilot study.

| Figure S1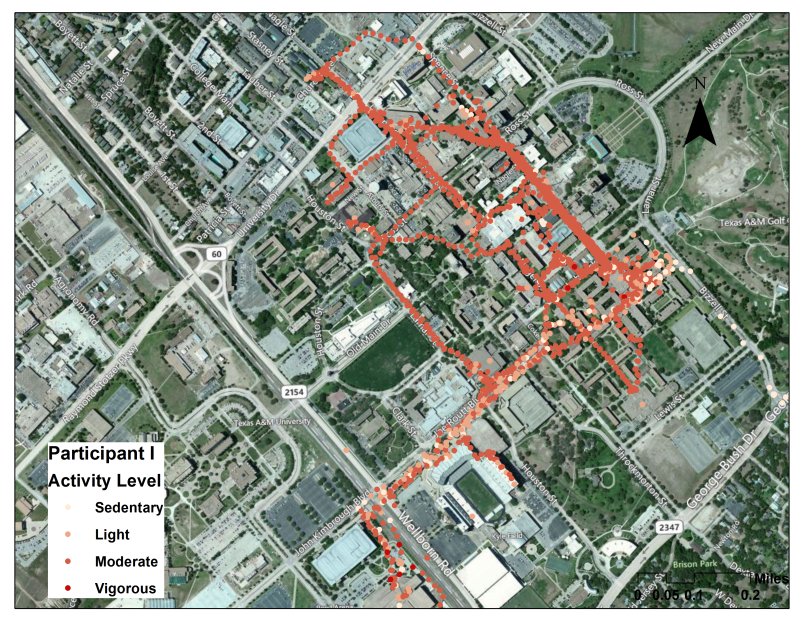 | Figure S2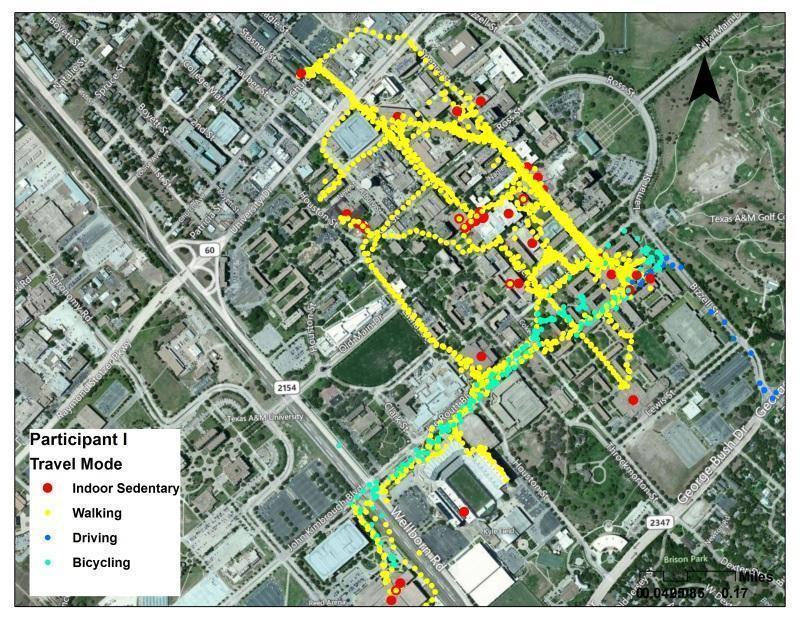 |
| --- | --- |

Although promising, these two tools also bear some inherent limitations.

First, the battery life and storage capacity of GPS have been major restraints. Currently, technical advances improve the devices a lot in both aspects, but those issues are still important considerations during the data collection process.

Second, the accuracy of GPS data is still somewhat questionable. For large-scale research, current wearable devices could generally meet the spatial accuracy requirement. But for most of the neighborhood studies, especially in an urban area, the distorted locational information may be problematic. In addition, some technical limitations have also been reported, such as cold/warm start or sensitivity to weather conditions.

Third, wearing both GPS and accelerometer devices for an entire week may be inconvenient for some participants.

More thorough assessments of GPS devices in terms of their applications in population-based PA research are reported in a journal paper (Wieters M, Kim J, Lee C, 2011). Assessment of Wearable Global Positioning System Units for Physical Activity Research. Journal of Physical Activity and Health). Based on many years of experience with these devices, our team has developed a systematic methodology to process and analyze the GPS/accelerometer data, from data cleaning and data synthesis to parameter computing, travel mode detection as well as travel trip identification, utilizing ArcGIS and Matlab (Figure S3 & Table S2).

Figure S3: Flowchart of Data Synthesis

**
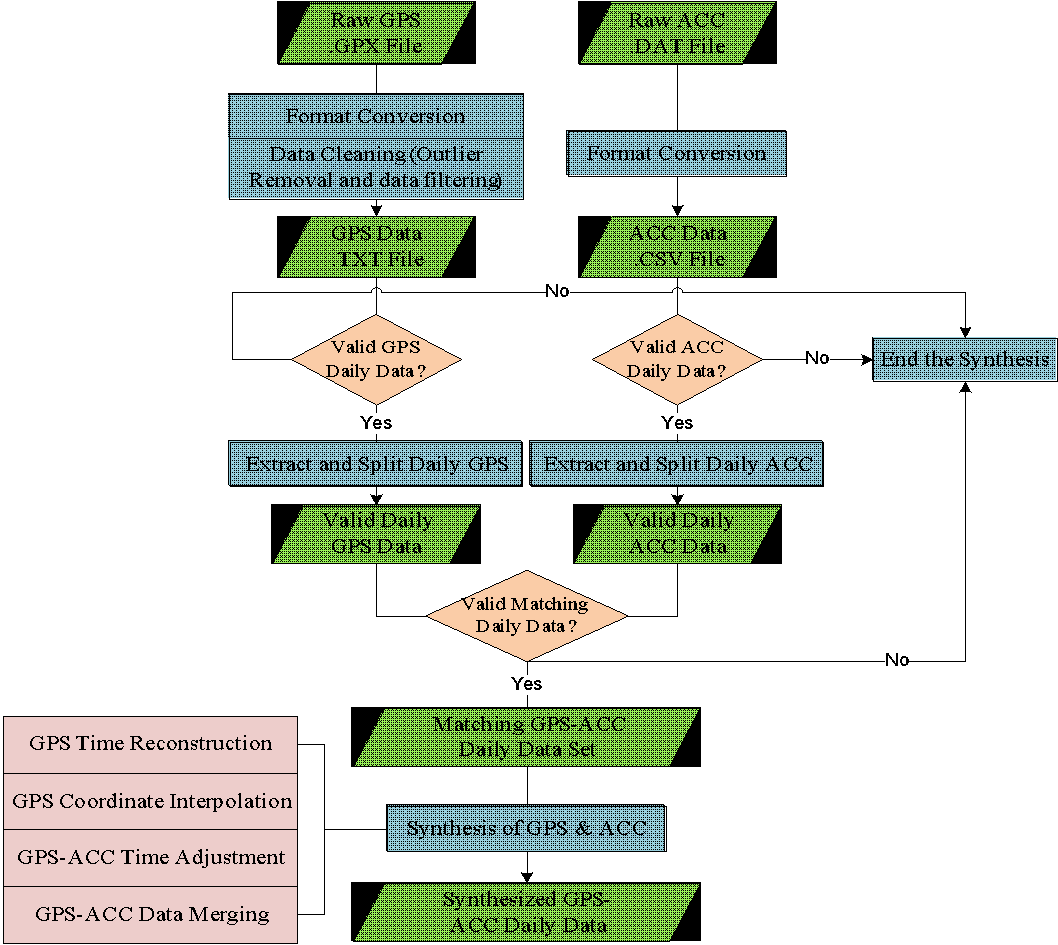
**

Table S2: Criteria of Travel Mode Detection

|  | Step (Low) | Step (High) | Activity (Light) | Activity (Moderate) | Activity (Vigorous) | Speed (walk) | Course_Std | Course_Change | *ValidGPS* | *Speed* |
| --- | --- | --- | --- | --- | --- | --- | --- | --- | --- | --- |
| Walk |  | ✔ |  | ✔ |  | 🗹 | 🗹 | 🗹 |  |  |
| Indoor Walk or Missing GPS |  | ✔ |  | ✔ |  |  |  |  | 🗶 |  |
| Walk with possible GPS |  | ✔ |  | ✔ |  |  |  |  | ✔ |  |
| Walk missing ACC |  | 🗶 |  | 🗶 |  | ✔ | 🗹 | 🗹 |  |  |
| Running |  | ✔ |  |  | ✔ |  |  |  |  |  |
| Driving | 🗶 | 🗶 |  |  |  |  | 🗹 | 🗹 | ✔ | >10 |
| Biking | 🗹 | 🗶 | 🗹 |  |  |  | 🗹 | 🗹 | ✔ | >5 |
| Slow walk |  | ✔ | ✔ |  |  |  |  |  |  |  |
| Other Indoor Activity | 🗹 |  | 🗹 |  |  |  |  |  |  |  |

‘✔’ & ‘🗶’: Necessary positive and negative conditions;

‘🗹’: Possible positive conditions
